# Unmasking orthostatic hypotension after cellular therapy: risk factors, implications, and clinical significance

**DOI:** 10.1007/s00520-026-11247-z

**Published:** 2026-09-26

**Authors:** Jorge Raul Vazquez-Urrutia, Jessica Santucci, Puneet Modgil, Amar Lal, Yoshitaka Inoue, Jenny Zimmerman, Rachel Woodworth, Karen Knol, Emily Hess, Julianne Jordan, Julia Stover, Emily Rekawek, Joseph Cioccio, Kevin Rakszawski, Natthapol Songdej, Myles Nickolich, Hong Zheng, Seema Naik, Christopher Ehmann, Brooke Silar, Caitlin Vajdic, Denise Bartram, Shin Mineish, Kentaro Minagawa

**Affiliations:** 1 Blood and Marrow Transplant Program, Division of Hematology and Oncology, Penn State Cancer Institute, 500 University Dr., Hershey, PA USA; 2 Penn State College of Medicine, Hershey, PA USA; 3 Department of Hematology, Kumamoto University, Kumamoto, Japan; 4 Penn State Cancer Institute Inpatient Unit, Penn State Health Hershey Medical Center, Hershey, PA USA; 5 Penn State Cancer Institute Outpatient Unit, Penn State Health Hershey Medical Center, Hershey, PA USA

**Keywords:** Orthostatic hypotension, Falls, Cellular therapy, Hematopoietic cell transplantation, Chimeric antigen receptor T-cell therapy

## Abstract

**Purpose:**

Orthostatic hypotension (OH) is a common complication in cancer care, increasing the risk of falls. However, in hematopoietic cell transplantation (HCT) and cellular therapy, few studies have focused on OH, and its incidence and risk factors remain unclear.

**Methods:**

This single-center retrospective study included 297 (autologous, 153; allogeneic, 127; and CAR-T, 17) patients. Blood pressure and heart rate were measured with the patient supine, and again at 1 min and at 3 min after standing. Data were collected to calculate OH incidence by day 30 post-infusion.

**Results:**

The cumulative incidence of any OH by day 30 was 57.6% (median onset, day 5), with symptomatic OH at 21.9% (median onset, day 4). Stratified by therapy type, the cumulative incidence of any and symptomatic OH at day 30 were 62.8% and 28.8% in the auto-HCT, 52.0% and 15.7% in the allo-HCT, and 52.9% and 5.9% in the CAR-T group, respectively. Multivariate analysis identified outpatient treatment (HR = 3.60, *p* < 0.01) and prior OH history (HR = 1.76, *p* < 0.01) as significant risk factors for any OH. For symptomatic OH, outpatient status (HR = 4.66, *p* < 0.01), > 2% weight loss (HR = 2.09, *p* = 0.02), and infection (HR = 2.57, *p* = 0.01) were associated with higher risk.

**Conclusion:**

It is important to assess OH before and after cellular therapy. In addition, daily weight monitoring and optimal fluid management may reduce the risk of OH development and lead to improved patient care and quality of life.

**Supplementary Information:**

The online version contains supplementary material available at https://doi.org/10.1007/s00520-026-11247-z.

## Introduction

Orthostatic hypotension (OH) is classically defined as a decrease in systolic blood pressure of 20 mmHg or more or a decrease in diastolic blood pressure of 10 mmHg or more upon standing [1, 2]. While some patients remain asymptomatic, others may experience sensations of dizziness, lightheadedness, or visual disturbances. In certain cases, OH can also lead to symptoms such as fatigue, difficulty concentrating, or neck discomfort [3]. Risk factors such as advanced age (> 70 years), neurodegenerative disorders with autonomic dysfunction, peripheral neuropathy, and prior syncopal episodes are associated with increased incidence of this phenomenon [4]. Furthermore, several studies have reported an association between OH and falls, which is a major safety and quality of care concern [5–7].

In the field of cellular therapies, including hematopoietic cell transplantation (HCT) and chimeric antigen receptor T-cell (CAR-T) therapy, the evaluation and management of OH are also important aspects of patient care. For instance, a retrospective study in a 56-patient cohort that underwent CAR-T demonstrated a 79% incidence of OH within 48 h after discharge, with older age and lower blood pressure being linked to this outcome [8]. Similarly, another study reported a 64% incidence of OH in multiple myeloma patients undergoing autologous-HCT (auto-HCT) within 7 days post-transplant, where ≥ 0.5% weight loss/day, white race, gabapentin, and antihypertensives were described as possible risk factors [9]. Another study of 132 auto-HCT or allogeneic-HCT (allo-HCT) recipients showed a lower incidence of OH at 23%, with no clear risk factor association on multivariate analysis [10]. However, studies on OH in the field of cellular therapies remain limited. Consequently, the true incidence, risk factors, and the impact of OH on clinical outcomes of cellular therapies remain largely unclear. OH is associated with substantial morbidity, an increased risk of falls, and higher mortality in the general population [11], and its prevalence is elevated among patients with cancer [12, 13].

Given the limited understanding of the incidence and risk factors of OH in cellular therapy patients, we conducted this study to further investigate the issue. Specifically, we aimed to assess differences based on the type of treatment (auto-HCT, allo-HCT, and CAR-T) and inpatient or outpatient status, as well as the impact of treatment-related weight loss on OH development given its association with orthostatic intolerance in the general population [14].

## Methods

### Patients and study design

This retrospective study included patients hospitalized at Penn State Health Milton S. Hershey Medical Center for cellular therapies (auto-HCT, allo-HCT, or CAR-T) between January 2019 and December 2022. To compare OH occurrence between inpatient and outpatient populations, we later included patients who underwent outpatient transplantation between July and December 2024. Patients lacking OH assessments or onset dates were excluded. The Penn State Cancer Institute institutional review board approved this study (STUDY00020839), and it was conducted in accordance with the Declaration of Helsinki.

### Measurement and definition of OH

Blood pressure and heart rate were measured with the patient supine, and again at 1 min and at 3 min after standing; per institutional protocol, the standing measurement was obtained within a 3- to 5-min window when a reading at exactly 3 min was not feasible. OH was diagnosed if systolic blood pressure decreased by ≥ 20 mmHg or fell below 90 mmHg, and diastolic pressure dropped by ≥ 10 mmHg [15]. A heart rate increase of ≥ 20 bpm was not used as a diagnostic criterion, because heart rate is not part of standard consensus definitions of OH, and the postural tachycardia syndrome was not formally assessed. Syncope at the time of an orthostatic measurement was also captured; no syncopal events were documented in this cohort. Data were collected to calculate OH incidence by day 30 post-infusion. Symptomatic OH was defined as OH with one or more of the following symptoms at onset: nausea/vomiting, fatigue, dizziness/lightheadedness, palpitations, and headache. Patients who developed OH were generally managed with oral or intravenous fluid administration. All inpatients with OH were equipped with bed sensors as standard practice to prevent falls. Blood pressure and other vital signs, including orthostatic measurements, were obtained using a Philips IntelliVue MX500 patient monitor (Philips, Amsterdam, the Netherlands); orthostatic vital signs were in some cases obtained using a Philips EarlyVue VS30 monitor. For fall prevention, inpatients were monitored using Stryker IsoTour bed-exit alarms (Model 2872; Stryker, Kalamazoo, MI, USA), and Posey chair alarms were used for patients seated in chairs.

### Covariate definitions

We reviewed medical records for histories and clinical variables previously associated with OH development, including hypertension, smoking, diabetes, hypercholesterolemia, chronic kidney disease, coronary artery disease, heart failure, atrial fibrillation, heart valve disease, pericarditis, neuropathy, reduced cardiac function (by ejection fraction), and antihypertensive medication use [4]. A prior history of OH was defined as a documented diagnosis of OH in the medical record, or a pre-treatment orthostatic measurement meeting the blood pressure criteria above, recorded before cellular therapy. Antihypertensive medication use was assessed as the presence or absence of any antihypertensive agent at baseline.

Baseline weight was defined as that on day −2 prior to cell infusion, or day −3 if unavailable, to avoid confounding from fluid loading typically started on day −1. A > 2% weight loss threshold was chosen because weight loss exceeding 2% is generally recognized as an indicator of dehydration and has also been reported as a marker associated with reduced exercise performance [16]. The onset of weight loss was defined as the first day on which a > 2% reduction from baseline was documented post-infusion. Similarly, the onset of diarrhea or any infection within day 30 was defined as the first day the respective condition was observed. Infection was defined as any clinically or microbiologically documented infectious event regardless of site or pathogen, including neutropenic fever, bloodstream, respiratory, gastrointestinal, urinary, and other infections; bacterial, viral, and fungal etiologies were all included.

### Statistical methods

For group comparisons, Fisher’s exact test was used for categorical variables, and the Mann–Whitney *U* test was used for continuous variables. OH development and death within day 30 were treated as competing risks. For symptomatic OH analysis, asymptomatic OH within day 30 was treated as a competing event. Cumulative incidences of OH were estimated using competing risks models, and differences between groups were assessed with Gray’s test. To identify independent predictors of OH development, univariate and multivariate analyses were conducted using the Fine and Gray proportional subdistribution hazards model. The covariates used in the univariate and multivariate analyses are listed in the table. The following variables were treated as time-dependent covariates in the analyses: weight loss > 2% from baseline within day 30 post-infusion, development of diarrhea within day 30 post-infusion, and development of any infectious disease within day 30 post-infusion. We also evaluated the impact of OH on outcomes, including relapse, non-relapse mortality (NRM), and overall survival (OS) beyond day 30. Relapse and NRM were treated as competing events, and cumulative incidence functions were plotted and compared using Gray’s test. The OS was estimated using the Kaplan–Meier method and was compared with the log-rank test. All statistical tests were two-sided, with *p* < 0.05 considered significant. Analyses were performed using EZR version 1.68 (Saitama Medical Center, Jichi Medical University, Saitama, Japan) [17].

## Results

### Patient characteristics

The study included a total of 297 patients (278 hospitalized, 19 outpatients). Of these, 153 patients were in the auto-HCT group, 127 in the allo-HCT group, and 17 in the CAR-T group. Among the auto-HCT patients, 115 (75.2%) had plasma cell disorders, and 34 (22.2%) had lymphomas. Among the allo-HCT patients, the most common diagnosis was AML in 45 patients (35.4%), followed by MDS/MPN in 35 patients (27.6%), all in 17 patients (13.4%), and lymphoma in 15 patients (11.8%). Only seven allo-HCT patients (5.5%) had plasma cell disorders. All CAR-T patients had lymphoma, and cytokine release syndrome occurred in five patients (grade I, 2; grade II, 3). All outpatients had plasma cell disorders and received auto-HCT.

Of the entire cohort, 126 patients did not develop OH (non-OH group), while 171 patients developed OH (OH group) within day 30 post-infusion. Patient characteristics are summarized in Table 1. Compared to the non-OH group, the OH group had significantly more patients with prior OH history (*p* = 0.048). In the outpatient setting, 18 of the 19 patients developed OH. There were no significant differences between the two groups in other patient characteristics, including factors that have been reported as risk factors for OH.

**Table 1:** Patient characteristics

Number of patients		Non-OH post-HCT	OH post-HCT	*p*-value
		126	171	
Age at HCT	Median [range], years	63 [21, 75]	62 [22, 77]	0.942
Sex (%)	Female	49 (38.9)	68 (39.8)	0.905
	Male	77 (61.1)	103 (60.2)	
Disease (%)	AML	23 (18.3)	22 (12.9)	0.465
	ALL	9 (7.1)	8 (4.7)	
	MDS/MPN/CML	13 (10.3)	22 (12.9)	
	Lymphoma	25 (19.8)	41 (24.0)	
	Plasma cell disorder	49 (38.9)	73 (42.7)	
	Other	7 (5.6)	5 (2.9)	
KPS (%)	≥ 80	42 (33.3)	67 (39.2)	0.331
	< 80	84 (66.7)	104 (60.8)	
HCT-CI (%)	0–1	23 (18.3)	31 (18.1)	1.000
	≥ 2	102 (81.0)	139 (81.3)	
	Unknown	1 (0.8)	1 (0.6)	
HCT type (%)	Auto	57 (45.2)	96 (56.1)	0.184
	Allo	61 (48.4)	66 (38.6)	
	CAR-T	8 (6.3)	9 (5.3)	
History of OH pre-HCT (%)	No	87 (69.0)	101 (59.1)	0.048
	Yes	36 (28.6)	69 (40.4)	
	Unknown	3 (2.4)	1 (0.6)	
History of hypertension (%)	No	56 (44.4)	79 (46.2)	0.814
	Yes	70 (55.6)	92 (53.8)	
Ventricular EF before HCT (%)	< 60%	28 (22.2)	41 (24.0)	0.088
	≥ 60%	97 (77.0)	121 (70.8)	
	Unknown	1 (0.8)	9 (5.3)	
Antihypertensive drugs at HCT (%)	No	61 (48.4)	88 (51.5)	0.639
	Yes	65 (51.6)	83 (48.5)	
Any medical history except HT (%)	No	37 (29.4)	58 (33.9)	0.451
	Yes	89 (70.6)	113 (66.1)	
Inpatient/outpatient (%)	Inpatients	125 (99.2)	153 (89.5)	< 0.001
	Outpatients	1 (0.8)	18 (10.5)	

*ALL* acute lymphoblastic leukemia, *Allo* allogeneic hematopoietic cell transplantation, *AML* acute myeloid leukemia, *Auto* autologous hematopoietic cell transplantation, *CAR-T* chimeric antigen receptor T-cell therapy, *CML* chronic myeloid leukemia, *EF* ejection fraction, *HCT* hematopoietic cell transplantation/therapy, *HCT-CI* hematopoietic cell transplantation–comorbidity index, *HT* hypertension, *KPS* Karnofsky performance status, *MDS* myelodysplastic syndromes, *MPN* myeloproliferative neoplasms, *OH* orthostatic hypotension

### Incidence of OH in cellular therapy

**Fig. 1:**
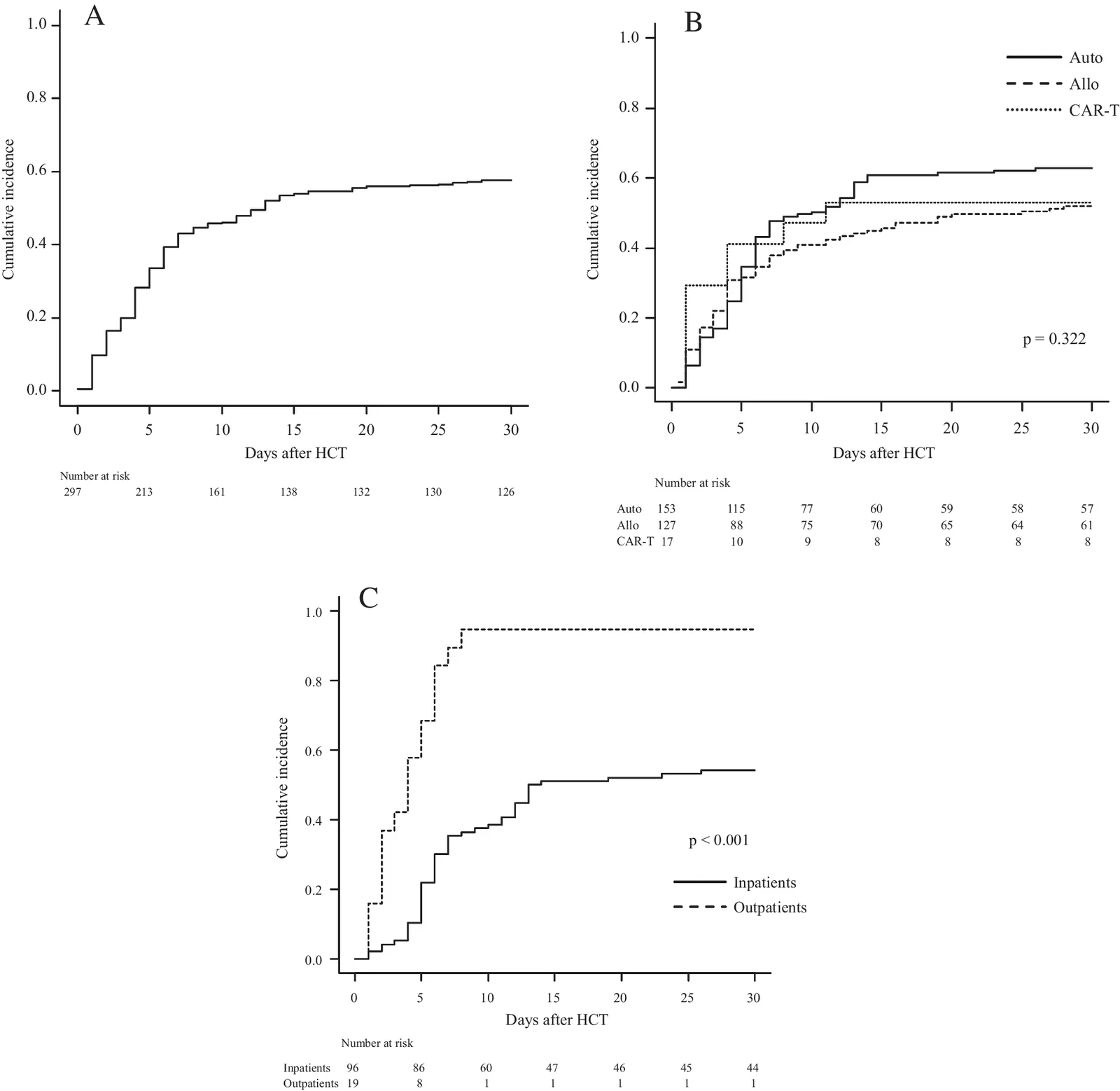
Cumulative incidence of all orthostatic hypotension. **A** All patients. **B** Comparison by hematopoietic cell transplantation/therapy type in all patients. The solid line represents auto-HCT patients, the dashed line represents allo-HCT patients, and the dotted line represents CAR-T patients. **C** Comparison between inpatients and outpatients among patients with autologous HCT and plasma cell disorders. The solid line represents inpatients, and the dashed line represents outpatients

Among all treatment groups, the cumulative incidence of OH was 53.9% at day 15 and 57.6% at day 30, with a median onset on day 5 (range, days 0–28) (Fig. 1A). When stratified by cellular therapy types, the cumulative incidence of OH at Day 30 was 62.8% in the auto-HCT group, 52.0% in the allo-HCT group, and 52.9% in the CAR-T group (*p* = 0.322; Fig. 1B). Although the incidence was similar across groups, the CAR-T group tended to have an earlier onset of OH compared to the other groups (median onset [range]: day 5 [days 1–26] in the auto-HCT group, day 4 [days 0–28] in the allo-HCT group, and day 1 [days 1–11] in the CAR-T group; *p* = 0.069). To assess differences in OH incidence by care setting, we analyzed 96 inpatients and 19 outpatients with plasma cell disorders who underwent auto-HCT. The incidence of OH was significantly higher in outpatients compared to inpatients (94.7% vs. 54.2% at day 30, *p* < 0.001; Fig. 1C). The median time to onset of OH was day 6 (range, days 1–26) in inpatients and day 4 (range, days 1–8) in outpatients, with significantly earlier onset in outpatients (*p* = 0.001).

**Fig. 2:**
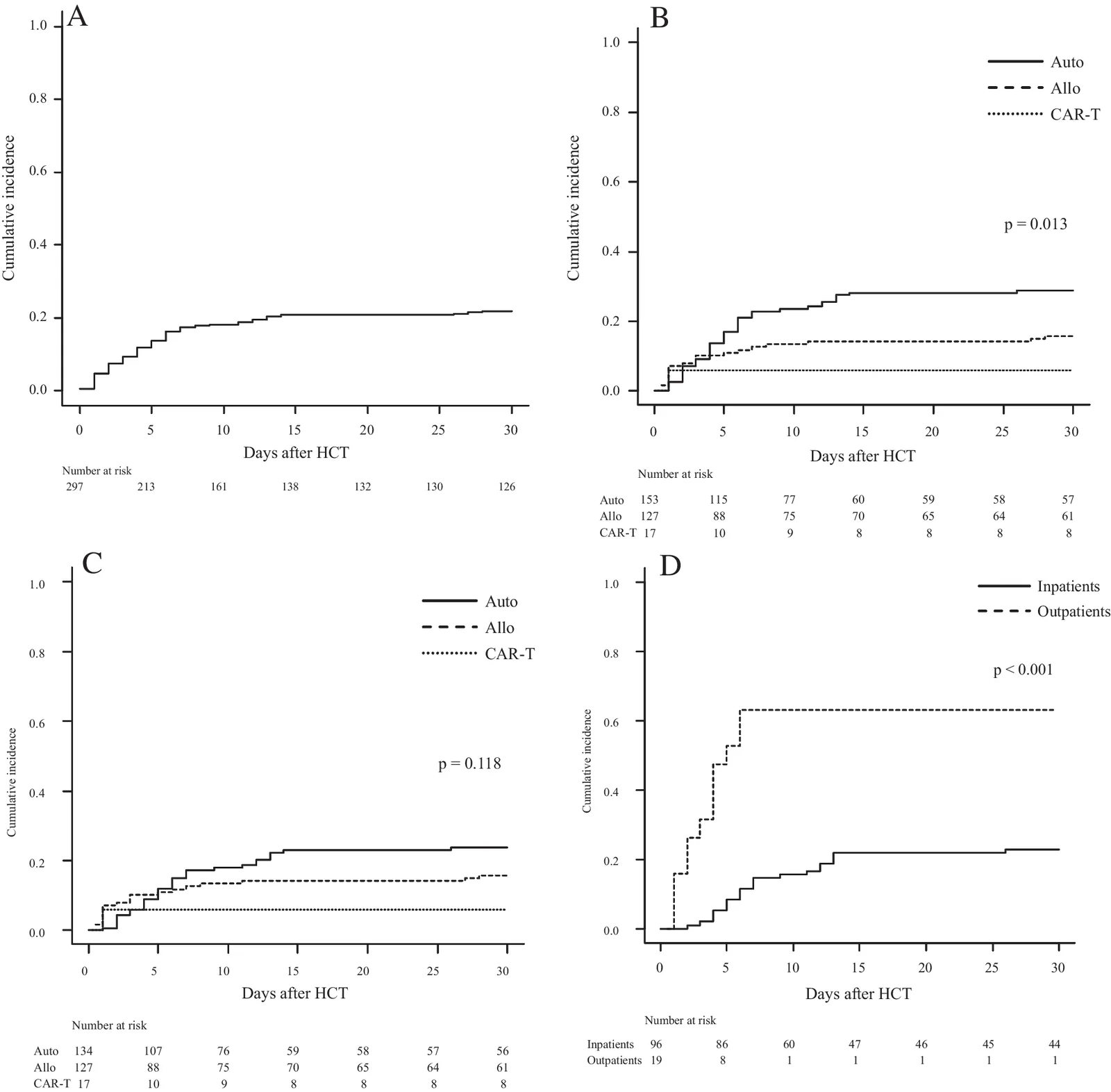
Cumulative incidence of symptomatic orthostatic hypotension. **A** All patients. **B** comparison by hematopoietic cell transplantation/therapy type in all patients. The solid line represents auto-HCT patients, the dashed line represents allo-HCT patients, and the dotted line represents CAR-T patients. **C** Comparison by hematopoietic cell transplantation/therapy type only in inpatients. The solid line represents auto-HCT patients, the dashed line represents allo-HCT patients, and the dotted line represents CAR-T patients. **D** Comparison between inpatients and outpatients among patients with autologous HCT and plasma cell disorders. The solid line represents inpatients, and the dashed line represents outpatients

Among the 171 patients who developed OH, 65 (38.0%) experienced symptomatic OH. Most common symptoms were nausea/vomiting (45), fatigue (22), dizziness/lightheadedness (21), palpitations (9), and headache (2). The cumulative incidence of symptomatic OH was 20.9% at day 15 and 21.9% at day 30, with a median onset on day 4 (range, days 0–28) (Fig. 2A). When comparing cellular therapy types, the cumulative incidence of symptomatic OH at day 30 was 28.8% in the auto-HCT group, 15.7% in the allo-HCT group, and 5.9% in the CAR-T group, with a significantly higher incidence observed in the auto-HCT group (*p* = 0.013; Fig. 2B). The median time to onset of symptomatic OH was day 5 (range, days 1–26) in the auto-HCT group, day 2 (range, days 0–28) in the allo-HCT group, and day 1 in the CAR-T group, indicating a trend toward later onset in the auto-HCT group (*p* = 0.058). Even when limited to hospitalized patients, although the difference was not statistically significant, the auto-HCT group showed a similar trend toward a higher incidence of symptomatic OH (*p* = 0.118; Fig. 2C). We compared patients with lymphoma and those with plasma cell disorders in the auto-HCT group. No significant difference was observed between the two disease types (Supplementary Fig. 1). Furthermore, among auto-HCT patients with plasma cell disorders, we compared the cumulative incidence of symptomatic OH between inpatients and outpatients. Consistent with the overall OH findings, the incidence of symptomatic OH was significantly higher in outpatients (63.2% vs. 22.9% at day 30, *p* < 0.001; Fig. 2D). The median time to onset was also significantly earlier in outpatients than in inpatients (day 3 [range, days 1–6] vs. day 6 [range, days 2–26], *p* = 0.001).

### Risk factors for OH occurrence

We performed univariate and multivariate analyses of patient characteristics, including factors previously reported as risk factors for OH (Table 2). In the multivariate analysis, a history of OH prior to cellular therapy (hazard ratio (HR), 1.76; 95% confidence interval (CI), 1.27–2.43; *p* < 0.01) and outpatient treatment (HR, 3.60; 95% CI, 2.31–5.61; *p* < 0.01) were identified as significant risk factors for any OH development. Additionally, a weight loss > 2% was associated with a trend toward increased risk of any OH (HR, 1.45; 95% CI, 0.99–2.11; *p* = 0.05).

**Table 2:** Univariate and multivariate analysis of factors predicting the development of orthostatic hypotension

Factors		All OH				Symptomatic OH			
		Univariate analyses		Multivariate analyses		Univariate analyses		Multivariate analyses	
		HR (95% CI)	*p*-value	HR (95% CI)	*p*-value	HR (95% CI)	*p*-value	HR (95% CI)	*p*-value
Age	< 60 years	1.00				1.00		1.00	
	≥ 60 years	0.94 (0.69–1.26)	0.66			0.52 (0.32–0.84)	< 0.01	0.38 (0.22–0.63)	< 0.01
Sex	Female	1.00				1.00		1.00	
	Male	1.01 (0.75–1.36)	0.94			0.62 (0.38–1.00)	0.05	0.56 (0.34–0.93)	0.02
Disease	Others	1.00				1.00		1.00	
	Plasma cell disorder	1.01 (0.76–1.35)	0.93			1.71 (1.05–2.76)	0.03	1.02 (0.49–2.12)	0.95
KPS	≥ 80	1.00				1.00			
	< 80	0.84 (0.63–1.13)	0.26			0.85 (0.52–1.39)	0.51		
HCT-CI	0–1	1.00				1.00			
	≥ 2	0.94 (0.64–1.4)	0.77			0.94 (0.5–1.78)	0.86		
HCT type	Auto	1.00				1.00		1.00	
	Allo	0.79 (0.58–1.07)	0.13			0.53 (0.31–0.90)	0.02	0.65 (0.30–1.42)	0.28
	CAR-T	0.91 (0.43–1.9)	0.80			0.19 (0.03–1.42)	0.11	0.21 (0.02–1.76)	0.15
History of OH pre-HCT	No	1.00		1.00		1.00			
	Yes	1.50 (1.11–2.03)	< 0.01	1.76 (1.27–2.43)	< 0.01	1.30 (0.79–2.14)	0.30		
History of hypertension	No	1.00				1.00			
	Yes	0.93 (0.69–1.24)	0.61			0.95 (0.59–1.54)	0.84		
Use of anti-hypertensive drugs	No	1.00				1.00			
	Yes	0.88 (0.66–1.17)	0.38			0.89 (0.55–1.43)	0.62		
Ventricular ejection fraction	< 60%	1.00				1.00			
	≥ 60%	0.90 (0.64–1.26)	0.54			1.22 (0.67–2.21)	0.52		
Any medical history except HT	No	1.00				1.00			
	Yes	0.86 (0.63–1.17)	0.33			0.90 (0.54–1.5)	0.68		
Inpatient/outpatient	Inpatients	1		1.00		1.00		1.00	
	Outpatients	3.15 (2.13–4.66)	< 0.01	3.60 (2.31–5.61)	< 0.01	4.80 (2.57–8.96)	< 0.01	4.66 (2.37–9.19)	< 0.01
> 2% weight loss from baseline*	No	1.00		1.00		1.00		1.00	
	Yes	1.43 (0.98–2.06)	0.06	1.45 (0.99–2.11)	0.05	1.98 (1.09–3.58)	0.02	2.09 (1.12–3.93)	0.02
Development of diarrhea within day 30*	No	1.00				1.00			
	Yes	1.01 (0.58–1.77)	0.96			1.08 (0.41–2.85)	0.88		
Development of any ID within day 30*	No	1.00				1.00		1.00	
	Yes	1.22 (0.73–2.02)	0.45			2.19 (1.00–4.78)	0.05	2.57 (1.26–5.24)	0.01

*Allo*, allogeneic hematopoietic cell transplantation; *Auto*, autologous hematopoietic cell transplantation; *CAR-T*, chimeric antigen receptor T-cell therapy; *CI*, confidence interval; *HCT*, hematopoietic cell transplantation/therapy; *HCT-CI*, hematopoietic cell transplantation–comorbidity index; *HR*, hazard ratio; *HT*, hypertension; *ID*, infectious disease; *KPS*, Karnofsky performance status; *OH*, orthostatic hypotension

^*^Time-dependent covariates

We next investigated the risk factors for the development of symptomatic OH. In the multivariate analysis, outpatient treatment (HR, 4.66; 95% CI, 2.37–9.19; *p* < 0.01), weight loss > 2% (HR, 2.09; 95% CI, 1.12–3.93; *p* = 0.02), and the occurrence of infections (HR, 2.57; 95% CI, 1.26–5.24; *p* = 0.01) were identified as significant risk factors. In contrast, older age (≥ 60 years; HR, 0.38; 95% CI, 0.22–0.63; *p* < 0.01) and male patients (HR, 0.56; 95% CI, 0.34–0.93; *p* = 0.02) were associated with a lower risk of developing symptomatic OH.

### Relationship between the occurrence of OH and fall events

**Fig. 3:**
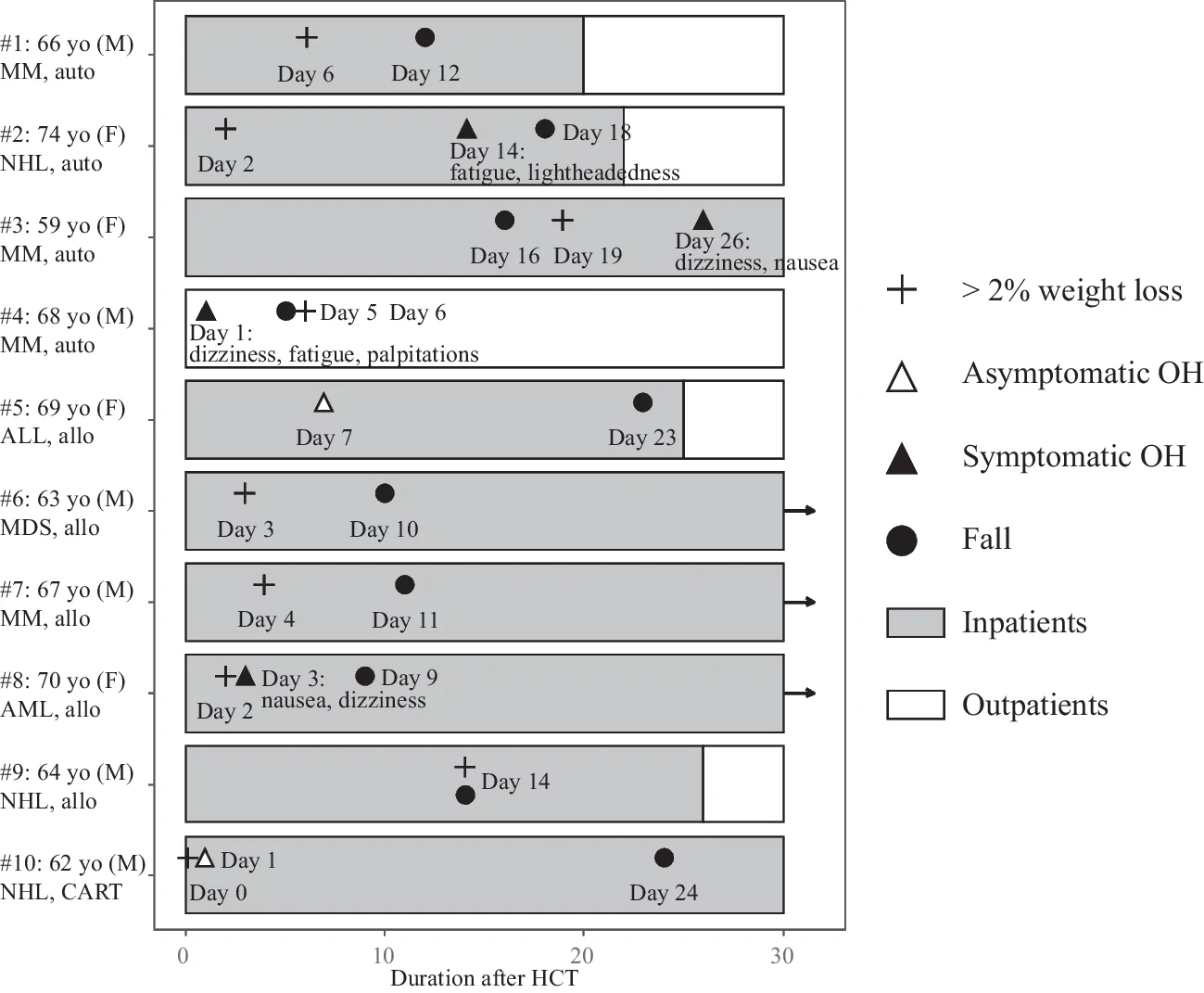
Clinical course of patients with falls. Illustrates the timeline and clinical events of patients who experienced falls after hematopoietic cell transplantation or CAR-T therapy. Each line represents the clinical course of an individual patient. Gray bars indicate inpatient periods, and white bars indicate outpatient status. The positive sign “ + ” indicates the occurrence of > 2% wight loss; the triangle “△” indicates the occurrence of asymptomatic orthostatic hypotension; the shaded triangle “▲” indicates the occurrence of symptomatic orthostatic hypotension, and the shaded circle “●” indicates the timing of falls. ALL, acute lymphoblastic leukemia; Allo, allogeneic hematopoietic cell transplantation; AML, acute myeloid leukemia; Auto, autologous hematopoietic cell transplantation; CAR-T, chimeric antigen receptor T-cell therapy; F, female; HCT, hematopoietic cell transplantation/therapy; M, male; MDS, myelodysplastic syndromes; MM, multiple myeloma; NHL, non-Hodgkin lymphoma; OH, orthostatic hypotension

Among the 297 patients, fall events within day 30 post-infusion were reported in ten patients. The clinical courses of these patients are illustrated in Fig. 3. Of the ten patients who experienced falls, four did not develop OH (#1, 6, 7, and 9). The remaining six developed OH (#2, 3, 4, 5, 8, and 10), of whom four (#2, 3, 4, and 8) developed symptomatic OH. However, one of the six (#3) developed OH only after the fall. There was no significant difference in the incidence of fall events between the non-OH and OH groups (*p* = 1.000). Similarly, there was no significant difference in the incidence of fall events based on the presence or absence of symptomatic OH (*p* = 0.234). Notably, nine out of the ten patients had a weight loss > 2% either before or shortly after the fall.

### Impact of OH occurrence on cellular therapy clinical outcomes

**Fig. 4:**
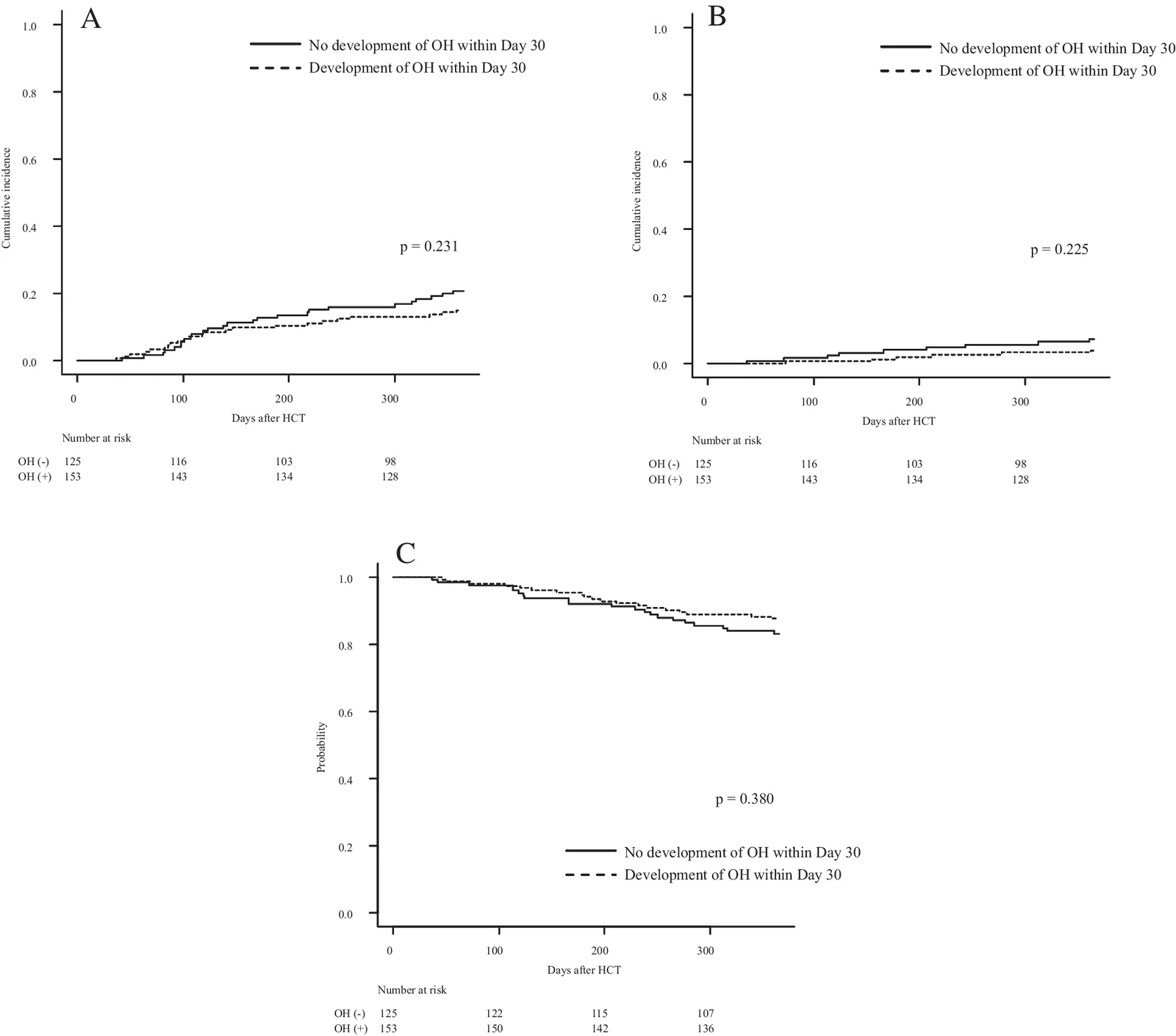
Impact of orthostatic hypotension on clinical outcomes in the inpatient cohort. **A** Cumulative incidence of relapse, **B** cumulative incidence of non-relapse mortality, and **C** probability of overall survival. The solid line represents patients without orthostatic hypotension within day 30, and the dashed line represents patients with any orthostatic hypotension within day 30

We assessed the impact of OH occurrence within day 30 post-infusion on subsequent clinical outcomes. Due to the shorter follow-up duration in outpatient cases, this analysis was restricted to the inpatient population (*N* = 278). The 100-day and 1-year relapse rates were 5.6% and 20.8% in the non-OH group, and 6.5% and 15.0% in the OH group, respectively (*p* = 0.231, Fig. 4A). The 100-day and 1-year NRM rates were 1.6% and 7.2% in the non-OH group, and 0.7% and 3.9% in the OH group, respectively (*p* = 0.225, Fig. 4B). The 100-day and 1-year OS rates were 97.6% and 83.2% in the non-OH group, and 98.0% and 86.9% in the OH group, respectively (*p* = 0.380, Fig. 4C). No statistically significant differences were observed between the groups in any of these outcomes. Subgroup analyses stratified by type of cellular therapy also showed no significant differences in outcomes according to OH status (data not shown). We further examined whether the presence of symptomatic OH had an impact on clinical outcomes, but symptomatic OH also had no impact on relapse, NRM, and OS (Supplementary Fig. 2A–C).

## Discussion

This is the largest study assessing the incidence, risk factors, and clinical impact of OH in patients undergoing cellular therapies. Our analysis revealed three major findings. First, more than half of the patients developed OH within day 30 post-infusion. Second, although the incidence of OH did not significantly differ between types of cellular therapy, the timing of onset varied slightly among groups. Third, a history of OH, the outpatient setting, body weight loss, and the development of infections might be risk factors for developing OH. Although the occurrence of OH was not directly associated with clinical outcomes, we believe that our findings provide valuable insights for patient care and maintaining quality of life (QOL) in cellular therapy settings.

More than half of the patients in our study developed OH within day 30 post-infusion. This finding is consistent with other studies, which have shown a similar incidence of OH in cellular therapies, ranging from 23 to 64% [8–10]. The wide range of incidence may relate to differences in study design. For example, the study by Patel et al. [8] had a limited follow-up of 48 h, while in the study by Ho et al. [9], data was recorded up to day 7 post-transplant. Conversely, Vecchié et al. [10] had a longer follow-up but with a lower incidence of OH after HCT of 23%, which could be explained by the fact that only symptomatic patients were accounted as OH patients. Interestingly, this incidence matches our finding of symptomatic OH in 21.9%. Notably, we demonstrated that the CAR-T patients had an earlier onset of OH compared to the other groups. This may be related to the increase in cytokines following treatment. These cytokines can lead to hypotension by increasing endothelial permeability and reducing the effective circulating volume [18]. Moreover, elevated levels of cytokines such as IL-6, TNF-α, and TGF-α are frequently observed in patients with advanced malignancy and have been correlated with disrupted circadian regulation and cortisol rhythms, implying impaired autonomic regulation [19]. In auto- and allo-HCT, several mechanisms may contribute to OH beyond the cytokine-mediated effects seen with CAR-T therapy. Conditioning-related gastrointestinal mucositis, nausea, and reduced oral intake frequently lead to intravascular volume depletion, while diarrhea and insensible losses further reduce effective circulating volume. Cumulative exposure to nephrotoxic and vasoactive agents, electrolyte disturbances, and the use of antiemetics and other medications with autonomic effects may also impair baroreflex-mediated compensation. In allo-HCT, additional contributors may include sepsis-associated vasodilation and, over the longer term, autonomic dysfunction related to the conditioning regimen or graft-versus-host disease.

We observed that a history of OH, outpatient status, and weight loss > 2%, and the occurrence of infection were associated with developing OH. Infections are often implicated in the development of distributive hypovolemia and shock, which could increase the risk of OH. This is the first study describing infection as a potential risk factor for OH in the cancer population. Among the identified infectious etiologies, we observed neutropenic fever as the most frequent cause in our cohort (21.6%). Furthermore, weight loss could exacerbate the hemodynamic instability associated with OH. Previous studies have shown that a lower BMI is associated with increased orthostatic intolerance [14]. Ho et al. [9] also reported that weight loss is a risk factor for the development of OH; however, they defined it as a weight loss of more than 0.5% per day. Further investigation is needed to determine the extent of weight loss that should be considered clinically significant. The increasing risk of OH in outpatients may reflect the challenges of outpatient management. From a patient perspective, this may be attributed to less fluid intake and poor nutritional status, while there is less monitoring and intensity of fluid management on the provider side. In order to mitigate this risk, we suggest that daily weight monitoring and preemptive fluid supplementation might help prevent OH occurrence, particularly in the outpatient setting. From a practical standpoint, OH can be readily detected in hematology patients by incorporating active orthostatic vital sign measurement—supine and standing blood pressure and heart rate—into routine assessment during and after cellular therapy, ideally at least daily during the highest-risk early post-infusion period and at each outpatient visit. Patients found to have OH can be managed with a stepwise approach that includes review and, where appropriate, temporary reduction of antihypertensive and other contributing medications, oral or intravenous volume repletion guided by daily weight, patient education on slow positional changes and adequate fluid and salt intake, and fall prevention measures such as bed-exit alarms for inpatients. Patients with symptomatic or recurrent OH, or those in the outpatient setting, may warrant closer monitoring and a lower threshold for preemptive fluid support. These practices are consistent with current cardio-oncology recommendations for the recognition and management of autonomic dysfunction, including OH, in patients with cancer [20, 21]. On the other hand, the finding that the risk of developing symptomatic OH decreased in older patients (≥ 60 years) warrants careful consideration. Generally, aging is associated with an increased risk of OH [3]. However, some studies have also reported that older adults often have diminished awareness of symptoms associated with reduced cerebral perfusion and may report falls rather than typical symptoms such as dizziness or syncope [22–24]. Therefore, close monitoring and thorough assessment are essential in this population. Importantly, this apparent inverse association between older age and symptomatic OH runs counter to the established literature, in which advancing age is generally associated with a higher risk of OH. We therefore interpret this finding with caution and consider it most likely to reflect bias rather than a true protective effect. Possible explanations include under-reporting and diminished symptom perception in older patients, ascertainment and detection bias inherent to a retrospective design, and confounding (for example, older patients may have been less likely to undergo outpatient therapy, which was itself the strongest risk factor for symptomatic OH). This observation should be regarded as hypothesis-generating and requires confirmation in prospective studies.

Our study had several limitations worth describing. First, although this is the largest study of OH in the field of cellular therapies, it is a single-center retrospective study, and the reporting of OH and OH-associated outcomes may be underrepresented. Second, OH assessment in outpatients began more recently, resulting in fewer cases and a limited scope of analysis. Third, because of the retrospective design, detailed data on several comorbidities relevant to OH—including Parkinson’s disease, thyroid disorders, and other autonomic conditions—and a complete class-level breakdown of antihypertensive medications were not consistently available, and these factors could not be fully adjusted for in the analyses. Fourth, the standing blood pressure measurement was obtained within a 3- to 5-min window rather than strictly at 3 min, which may have allowed inclusion of some delayed orthostatic responses. Fifth, the outpatient (*n* = 19) and CAR-T (*n* = 17) subgroups were small, so the corresponding analyses, including the strong outpatient effect, should be regarded as exploratory; the higher OH incidence observed in outpatients may partly reflect differences in blood pressure monitoring, hydration protocols, or detection rather than a purely biological effect, and between-group comparisons involving the CAR-T cohort do not support causal conclusions. Sixth, the > 2% weight loss threshold, although supported by prior data, is to some extent arbitrary; alternative definitions, such as the ≥ 0.5% per day threshold used by Ho et al. [9], could yield different estimates, and the optimal cutoff remains to be determined. Finally, our study did not demonstrate a clear association between the occurrence of OH and the incidence of falls. Given that only ten fall events were observed, the study was underpowered to detect such an association, and the absence of a statistically significant difference should not be interpreted as evidence of no association. In clinical practice, patients who developed OH often received oral or intravenous fluid supplementation, and among inpatients, bed sensors were routinely used as a fall prevention measure. These interventions may have contributed to reducing the incidence of falls even in patients with OH.

In conclusion, we observed that OH is highly prevalent in patients undergoing cellular therapies. In both outpatient and inpatient settings, it is important to assess OH before and after cellular therapy. In addition, daily weight monitoring and optimal fluid management may reduce the risk of OH development and lead to improved patient care and QOL. Further prospective studies are needed to validate these findings in cellular therapy patients.

## Supplementary Information

Below is the link to the electronic supplementary material.

ESM 1 (DOCX 617 KB)

## Data Availability

The datasets generated during and/or analyzed during the current study are available from the corresponding author upon reasonable request.

## Declarations

### Use of artificial intelligence (AI)-assisted technologies

We would like to disclose that in the preparation of our manuscript we have used Artificial Intelligence (AI) tools (ChatGPT-4 Omni and Claude Opus 4.8) for language editing and grammar improvement. The authors have reviewed and approved all AI-assisted edits to ensure the accuracy and integrity of the content.
