# Supplementary material for: Unmasking orthostatic hypotension after cellular therapy: risk factors, implications, and clinical significance
Source: Support Care Cancer. 2026 Sep 26;34(10):1023. doi: 10.1007/s00520-026-11247-z (PMC13615906; doi:10.1007/s00520-026-11247-z)
Supplement: Supplementary file 1 [file 520_2026_11247_MOESM1_ESM.docx]

**Supplementary Figure 1. Cumulative incidence of symptomatic OH comparing disease (Only in auto-HSCT patients).**

**Supplementary Figure 2 (inpatient cohort).**

(A) **Cumulative incidence of relapse.**

(B) **Cumulative incidence of non-relapse mortality.**

(C) **Probability of overall mortality.**
